# Triad of Myelinated Retinal Nerve Fibers, Axial Myopia and Amblyopia

**Published:** 2010-10

**Authors:** Jalil Naghib

**Affiliations:** Negah Eye Center, Tehran, Iran

**Dear Editor,**

I read with interest the recently published article titled “Unilateral Myelinated Retinal Nerve Fiber Layer Associated with Axial Myopia, Amblyopia and Strabismus” by Moradian and Karimi.^1^ I would like to share my experience with two similar cases.

## Case 1

A 4 year-old girl was referred because of refractory amblyopia in her right eye. Cycloplegic refraction was −8.00–3.00×180º and +1.00–1.50×10º in the right and left eyes respectively. Best corrected visual acuity was counting fingers at 4 meters in the right eye and 20/25 in the left eye. There was right esotropia of 20 prism diopters (PD). Slitlamp examination and pupillary light responses were normal. Funduscopic examination was normal in the left eye but showed myelination of the nerve fiber layer superotemporal and inferotemporal to the macula in the right eye. The peripapillary area and the fovea were not involved. The optic disc was hypoplastic and the foveal reflex was dull. Axial length was 26.62 and 22.12 mm in the right and left eyes respectively. Optical coherence tomography (OCT) of the myelinated area revealed increased thickness of the nerve fiber layer (NFL) to nearly 50% of total retinal thickness together with atrophy of underlying retinal layers. Due to disc hypoplasia, a brain and orbital MRI was requested, which was normal.

## Case 2

A 1 year-old girl presented with history of strabismus since birth. Refraction was +1.50–0.50×180º and −9.00–2.00×180º in the right and left eyes respectively. On examination, there was left exotropia of about 20 PD, localized posterior lens opacity, extensive myelination of peripapillary and macular nerve fibers, and a fibrovascular stalk arising from the superotemporal vascular arcade to the posterior lens capsule at the site of the opacity. No further investigations were conducted for this subject.

Different theories have been proposed to explain the pathogenesis of myelinated nerve fibers. It has been suggested that a defect in the lamina cribrosa might allow myelin to be deposited in the retina or adjacent to the optic disc but such a defect has not been demonstrated in autopsied eyes. Another explanation is that heterotopic oligodendrocytes or glial cells within the retina are responsible for abnormal myelination of retinal nerve fibers. This theory has been supported by the presence of glial cells resembling oligodendrocytes which are responsible for normal myelination in the central nervous system.^2^

Unilateral, and rarely bilateral, retinal myelination may be associated with high axial myopia, deep amblyopia, and strabismus.^3^–^5^ Ellis and coworkers^3^ reported 6 patients with myelinated NFL, high axial myopia and refractory amblyopia, and evaluated them with particular emphasis on sensory status. The authors suggested that low vision in these patients has an organic etiology in addition to functional amblyopia. They also suggested myelination around the macula to be the most likely cause of poor vision and proposed that glial cells or myelin impede transmission of light through the retina or impulses from the retina to the lateral geniculate body.

Associations reported with this syndrome include anterior segment abnormalities, congenital cataracts, choroidal and optic disc colobomas, persistent hyaloid membrane or artery, macular pucker, abnormal foveal reflex and macular pigmentary changes, decrease in ganglion cells, epilepsy, dolichocephaly and Von-Recklinghausen disease.^3^–^5^ The first case described herein had optic disc hypoplasia and the second patient had localized posterior lens opacity and a fibrovascular stalk attached to the posterior lens capsule at the site of the opacity.

Although the amblyopia in these patients has been called refractory, it consists of two components; one is the organic defect in the retina which is not treatable, but the other is due to anisometropia which does respond to amblyopia therapy and should therefore be corrected.
